# Care Outcomes for Chiropractic Outpatient Veterans (COCOV): a qualitative study with veteran stakeholders from a pilot trial of multimodal chiropractic care

**DOI:** 10.1186/s40814-021-00962-5

**Published:** 2022-01-14

**Authors:** Stacie A. Salsbury, Elissa Twist, Robert B. Wallace, Robert D. Vining, Christine M. Goertz, Cynthia R. Long

**Affiliations:** 1 Palmer Center for Chiropractic Research, Palmer College of Chiropractic, 741 Brady Street, Davenport, Iowa 52803 USA; 2 Department of Epidemiology, College of Public Health, The University of Iowa, S422 CPHB, 145 N. Riverside Drive, Iowa City, Iowa 52242 USA; 3 Department of Orthopaedic Surgery, Duke University School of Medicine, 200 Morris Street, Durham, North Carolina 27701 USA

**Keywords:** Veterans health, Stakeholder participation, Qualitative research, Health services, Health communication, Chiropractic, Low back pain, Data collection, Patient-reported outcome measures, Pilot study

## Abstract

**Background:**

Low back pain (LBP) is common among military veterans seeking treatment in Department of Veterans Affairs (VA) healthcare facilities. As chiropractic services within VA expand, well-designed pragmatic trials and implementation studies are needed to assess clinical effectiveness and program uptake. This study evaluated veteran stakeholder perceptions of the feasibility and acceptability of care delivery and research processes in a pilot trial of multimodal chiropractic care for chronic LBP.

**Methods:**

The qualitative study was completed within a mixed-method, single-arm, pragmatic, pilot clinical trial of chiropractic care for LBP conducted in VA chiropractic clinics. Study coordinators completed semi-structured, in person or telephone interviews with veterans near the end of the 10-week trial. Interviews were audiorecorded and transcribed verbatim. Qualitative content analysis using a directed approach explored salient themes related to trial implementation and delivery of chiropractic services.

**Results:**

Of 40 participants, 24 completed interviews (60% response; 67% male gender; mean age 51.7 years). Overall, participants considered the trial protocol and procedures feasible and reported that the chiropractic care and recruitment methods were acceptable. Findings were organized into 4 domains, 10 themes, and 21 subthemes. Chiropractic service delivery domain encompassed 3 themes/8 subthemes: scheduling process (limited clinic hours, scheduling future appointments, attendance barriers); treatment frequency (treatment sufficient for LBP complaint, more/less frequent treatments); and chiropractic clinic considerations (hire more chiropractors, including female chiropractors; chiropractic clinic environment; patient-centered treatment visits). Outcome measures domain comprised 3 themes/4 subthemes: questionnaire burden (low burden vs. time-consuming or repetitive); relevance (items relevant for LBP study); and timing and individualization of measures (questionnaire timing relative to symptoms, personalized approach to outcomes measures). The online data collection domain included 2 themes/4 subthemes: user concerns (little difficulty vs. form challenges, required computer skills); and technology issues (computer/internet access, junk mail). Clinical trial planning domain included 2 themes/5 subthemes: participant recruitment (altruistic service by veterans, awareness of chiropractic availability, financial compensation); and communication methods (preferences, potential barriers).

**Conclusions:**

This qualitative study highlighted veteran stakeholders’ perceptions of VA-based chiropractic services and offered important suggestions for conducting a full-scale, veteran-focused, randomized trial of multimodal chiropractic care for chronic LBP in this clinical setting.

**Trial registration:**

ClinicalTrials.gov NCT03254719

**Supplementary Information:**

The online version contains supplementary material available at 10.1186/s40814-021-00962-5.

## Key messages regarding feasibility

- What uncertainties existed regarding feasibility?

While chiropractic services are provided within VA healthcare facilities, the acceptability and feasibility of conducting pragmatic randomized trials of multimodal chiropractic care for low back pain in this setting was unknown.

- What are the key feasibility findings?

Qualitative interviews were conducted with the veterans who enrolled in this pilot trial to evaluate the acceptability of research methods and clinical care delivery. Veterans make key recommendations about treatment scheduling, reasons for participant involvement, and patient communication needs. Interviews clarified the burden and relevance of outcome measures and ways to better facilitate online data collection. Veterans also provided insights into the delivery of chiropractic treatments for this patient population.

- What are the implications of the feasibility findings for the design of the main study?

Findings suggest pragmatic trials of chiropractic care are feasible in VA settings, and that the participant recruitment strategies, treatment scheduling processes, selected outcome measures, and online data collection procedures used in this pilot were acceptable to most veterans. Compensation for completion of qualitative interviews was added to the study protocol to improve participation in this important component of the main study.

## Background

Military veterans often wage long-term battles with the overlapping problems of chronic pain and mental illness following discharge from the armed services [1]. More than 50% of U.S. veterans experience chronic pain syndromes, with pain intensity rated as moderate to severe [2]. Chronic pain prevalence rates increase each year following deployment [3] and may be more prevalent in female veterans [4]. Musculoskeletal pain is especially common, with chronic low back pain (LBP) impacting 25% of veterans [1]. Concurrent with musculoskeletal pain, many veterans also report high rates of depression, anxiety, post-traumatic stress disorder (PTSD), and substance use disorder, among other mental health comorbidities [1, 5]. Veterans with co-occurring chronic musculoskeletal pain and mental health conditions have higher rates of healthcare service utilization and greater use of prescription psychotropic and pain medication, including opioids [5, 6].

In response, the U.S. Department of Veterans Affairs, Veterans Health Administration (VA) has instituted policy efforts to improve clinical pain management and combat the opioid epidemic. One prominent recommendation is to integrate non-pharmacological and complementary and integrative health (CIH) approaches into VA primary care, pain care, and mental health settings when sufficient evidence of safety and effectiveness exists [7–9]. Nine approaches have been prioritized for early integration, including psychological and behavioral therapies; exercise and movement therapies; manual therapies including spine and joint manipulation; and team-based, multimodal pain care [8]. Many veterans with pain-related conditions use these, and other, CIH approaches regularly, with utilization rates trending upwards and ranging from 1–52% by therapy and study [9–12].

Sufficient evidence currently exists on the safety and effectiveness of spinal manipulation, a central component of multimodal chiropractic care, to recommend this treatment for veterans with LBP and neck pain [13]. In addition, veterans who receive chiropractic care may be less likely to use opioid medications [14]. Currently, however, few prospective studies on chiropractic care for veterans are available on which to base study protocols for randomized controlled trials (RCTs) designed to determine dosage and treatment parameters [15, 16]. Veterans increasingly choose chiropractic care as a therapeutic option, with both the number of patients served and treatment visits completed annually rising steadily since services were introduced into VA healthcare facilities in 2004 [17, 18]. In tandem with efforts to improve pain management services, VA has proposed research agendas for non-pharmacological treatments for chronic musculoskeletal pain [13]. Crucial scientific priorities remain for chiropractic, including implementation research to facilitate clinical adoption and patient access; effectiveness trials for non-spine-related musculoskeletal conditions; and research conducted with underrepresented veteran populations, including older adults, women, and patients with comorbidities, such as mental health concerns [13].

Toward this agenda, our team conducted a mixed-method, single-arm, pragmatic, pilot clinical trial of multimodal chiropractic care for veterans with chronic LBP and with or without mental health comorbidity. The aim of the Care Outcomes for Chiropractic Outpatient Veterans (COCOV) pilot trial was to evaluate the feasibility, safety, and acceptability of multimodal chiropractic care for a veteran population. The objectives of this qualitative study were to (1) identify veteran perceptions of the acceptability and feasibility of the trial’s research processes and (2) report participant recommendations for chiropractic services in VA settings.

## Methods

### Design

The research design was a qualitative interview study nested within a single-arm, pilot clinical trial of multimodal chiropractic care for veterans with chronic LBP [19]. This qualitative study used a descriptive, phenomenological perspective to understand veterans’ experiences with the research methods used in this pilot study and their suggestions for chiropractic services offered within VA settings [20, 21]. Additional file 1 provides the Consolidated Criteria for Reporting Qualitative Studies (COREQ) checklist for this study [22].

### Clinical trial background

Clinical trial design and primary results are presented in a companion article (Long CR, Salsbury SA, Vining RD, Lisi AJ, Corber L, Twist EJ, Abrams T, Wallace RB, Goertz CM: Care Outcomes for ChiropraLong CR, Salsbury SA, Vining RD, Lisi AJ, Corber L, Twist EJ, Abrams T, Wallace RB, Goertz CM: Care Outcomes for Chiropractic Outpatient Veterans (COCOV): a single-arm, pragmatic, pilot trial of multimodal chiropractic care for U.S. veterans with chronic low back pain, Submitted). Briefly, eligible veterans received up to 10 weeks of chiropractic care to address chronic LBP and back-related disability. Participants received on average 4.5 treatment visits (described further in results). Multimodal chiropractic care consisted of spinal manipulative therapy, spinal mobilization, other manual therapies, active exercise, and lifestyle advice delivered by licensed doctors of chiropractic (DC). An integrative care pathway for veterans with LBP also was provided as a reference for treatment decisions and referrals to primary care and mental health providers [19]. Outcome measures were completed online via REDCap (Research Electronic Data Capture, Vanderbilt, Nashville, TN, USA) at baseline and weeks 3, 5, 7, and 10 [23]. Outcome measures included recommended biomedical and psychosocial parameters [24], with the Roland Morris Disability Questionnaire as the primary outcome [25]. Patient-reported outcomes included established questionnaires and select instruments from the National Institutes of Health (NIH) Patient-Reported Outcome Measurements Information System (PROMIS®) [26] and the Pain Assessment Screening Tool and Outcomes Registry (PASTOR) [27], which were piloted with the legacy measures to evaluate their usefulness in veteran populations.

### Setting

The setting was a 2-site chiropractic clinic located in the State of Iowa, within Veterans Integrated Service Network (VISN) 23 of the VA Midwest Health Care Network. VISN-23 serves more than 440,000 veterans in parts of 11 states in the midwestern United States, including Iowa. Chiropractic services were provided through the Chiropractic/Acupuncture Clinic in the Extended Care and Rehabilitation Service Line. At the time of this trial, one chiropractic clinic was co-located with a pain clinic at the Iowa City VA Health Care System in Iowa City, Iowa, while the second was co-located within primary care at a community-based outpatient clinic in Coralville, Iowa. Two licensed DCs employed by VA provided chiropractic care. Although acupuncture was available at these chiropractic clinics, this modality was not delivered in the trial. Veterans received usual medical and mental health services from their current providers, with a chiropractic integrated care pathway provided as a resource for clinical evaluation and interprofessional communication and referrals [19].

### Participants

Eligibility criteria for trial participation are described in a companion article (Long CR, Salsbury SA, Vining RD, Lisi AJ, Corber L, Twist EJ, Abrams T, Wallace RB, Goertz CM: Care Outcomes for ChiropraLong CR, Salsbury SA, Vining RD, Lisi AJ, Corber L, Twist EJ, Abrams T, Wallace RB, Goertz CM: Care Outcomes for Chiropractic Outpatient Veterans (COCOV): a single-arm, pragmatic, pilot trial of multimodal chiropractic care for U.S. veterans with chronic low back pain, Submitted). Briefly, veterans age 18 years and older who reported chronic LBP consistent with the NIH Task Force on Research Standards for Chronic Low Back Pain [24] definition (LBP of at least 3 months duration and pain on at least half the days in the past 6 months) were eligible for the trial. Potential interview subjects included all trial participants, with no exclusions to the interview. Our recruitment goal was 40 participants, the sample size chosen to adequately assess the feasibility of participant recruitment and retention strategies, as well as electronic data collection processes. The sample was to include at least 20% female participants in both the trial and interviews, as oversampling of women veterans is a recommendation from VA women’s health research experts [28]. Participant recruitment for the clinical trial (also described in the companion article) included provider referrals, chiropractic clinic patient screening, focused mailings based on key characteristics identified in the electronic health record, and standard direct recruitment with posters placed in VA health settings and veteran-centric organizations. Study coordinators telephoned trial participants to invite them to complete the qualitative interview.

### Data collection

Interviews were scheduled from 2 weeks before until 2 months after a participant completed the 10-week trial. Two study coordinators conducted the interviews, either in person at the VA chiropractic clinics or by telephone in a call originating from the Palmer Center for Chiropractic Research in Davenport, IA. Participants read information about the interview during the informed consent process and received a verbal overview of its procedures before the interview. A structured question list (Table 1) guided the interview, with topics focused on participant perspectives of the chiropractic intervention and service delivery [29] and the feasibility and acceptability of the clinical trial procedures, as guided by the CONSORT checklist [30]. Participants were encouraged to elaborate on aspects of the trial they found difficult or challenging to complete through probes on more closed-ended questions or when brief replies were offered. For example, follow-up questions asked participants about outcomes that were important to them, but which were not included on data collection forms. Interviews were recorded using digital recording devices and uploaded to a secure website for professional transcription (Way With Words, New York). Transcripts were reviewed against the audiorecordings to assess accuracy by the lead qualitative investigator.

**Table 1:** Interview questions

1) Tell me about your experience receiving chiropractic care in the VA. How did this care meet your expectations? How might we improve this care in the future?	
2) Did the forms you filled out ask about topics that are important to you?	
3) How much work or burden was filling out those forms for you?	
4) Were there forms that you didn’t think applied to your situation?	
5) What challenges did you have accessing the online study forms?	
6) What challenges did you have accessing MyHealth*e*Vet?	
7) How was the treatment schedule for you? That is, did you see the chiropractor too little, too much, or just about the right number of visits? Why was that?	
8) What changes should we make to the study to make it work better for veterans?	
9) Do you have any questions or any final comments about the study?	

### Data analysis

This qualitative content analysis used a directed approach to identify salient topics for the implementation of a full-scale randomized clinical trial of chiropractic care within VA [31], such as treatment scheduling, communication processes, outcome measures, data collection procedures, and chiropractic clinic recommendations [32]. The primary data analyst (SAS) achieved familiarity with the text through open reading of the complete transcripts, with successive transcript readings identifying discrete topics of interest aligning with key issues in clinical trial implementation. Meaning units were identified, with salient passages from the transcript transferred to a spreadsheet by participant identification number (PTID) and coding domain. Subsequent analysis rounds were organized into data tables to form patterns that included sub-coding within each domain to categorize emerging themes and subthemes. Coding continued until no new themes were identified. Data tables were provided to co-investigators and study staff for comment, clarification, and revision (see Additional files 1 and 2). Representative quotes were offered by PTID with names and/or gender removed whenever possible to allow anonymity of study participants and DCs.

### Ethical considerations

Ethics approvals were granted by the Palmer College Foundation Institutional Review Board (IRB), The University of Iowa IRB (IRB-03 VA Only), and the VA Connecticut Health System IRB. Participants provided written informed consent to enroll in the trial which included information about the qualitative component of the research. Veterans completed VA Authorization Form #10-0493 to allow the creation and use of an audiorecording for research purposes. Participants also gave verbal consent to the recording process before the interview. Participants received up to $100 in gift cards for completion of primary outcome measures collected via REDCap during the trial, although veterans received no additional incentive to complete the qualitative interview.

## Results

### Characteristics of the sample and interviews

Of 40 trial participants, 24 completed interviews (60% response). Male veterans (*n* = 16; 67%) predominated the interviews, although the inclusion of 8 women veterans (33%) as interview participants achieved our goal of a minimum 20% female sample. Mean age (SD) was 51.7 (15.7) years with most participants stating their race as white (88%) and their ethnicity as non-Hispanic or Latinx (96%). All participants (100%) stated chronic LBP was an ongoing problem for more than 6 months. As consistent with our eligibility criteria, which included veterans with or without selected mental health conditions, most participants (95%) had either a history of mental health comorbidity documented in their electronic health record or positively screened for depression, anxiety and/or post-traumatic stress disorder on their baseline outcomes.

Interview duration averaged 16:46 (range 6:42 to 42:37), with 11 conducted face-to-face at the VA chiropractic clinics and 13 completed as telephone interviews. Of the 16 participants who did not complete an exit interview, 5 were not able to be contacted by study staff while the remainder had scheduling conflicts, or the veteran decided to not participate.

### Overall findings

**Fig. 1:**
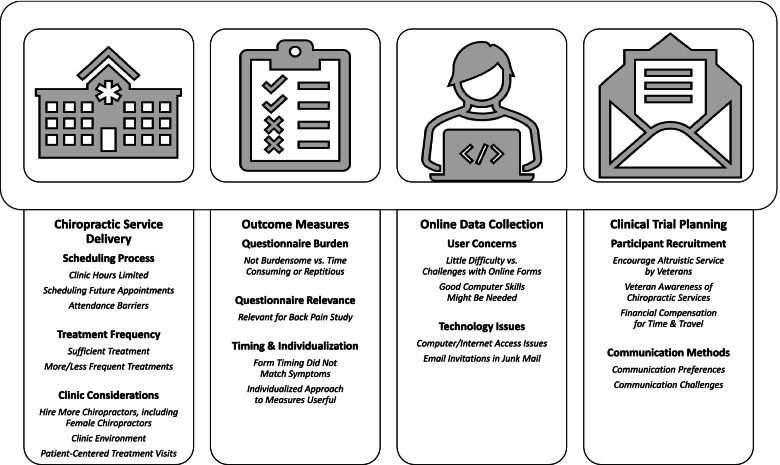
Qualitative themes from a pilot clinical trial of chiropractic care for veterans with low back pain

Study findings (Fig. 1) provided critical information for the planning of a full-scale, pragmatic randomized clinical trial of chiropractic care in VA settings that was subsequently funded by NIH [31]. Our results also offer a view into the patient perspective of the delivery of chiropractic care within VA. Domain 1 related to chiropractic service delivery in VA, emphasizing processes related to scheduling and attending chiropractic treatments. Domains 2 and 3 addressed methodological concerns for conducting chiropractic clinical trials within VA, highlighting patient perceptions of selected outcome measures and the logistics of the online data collection. Domain 4 offers insights into other clinical trial planning considerations, including participant recruitment and communication.

### Domain 1: Chiropractic service delivery for a clinical trial in VA

Domain 1 outlined veterans’ perspectives about the delivery of chiropractic services within a clinical trial conducted in VA settings. Themes included the scheduling process, treatment frequency, and patient-centered clinic environments, including personnel staffing.

#### Theme 1: Scheduling process for chiropractic services

Participants offered positive (*n* = 10) or mixed (*n* = 9) comments about their scheduling experiences, with DCs and clinic staff helping navigate the process. Negative comments (*n* = 5) pertained to unexpected delays in appointments, with some reporting waits of 2 weeks to 1 month.

##### Clinic hours limited

Veterans noted the VA clinic offered limited hours (8:00 a.m.–4:30 p.m.) and weekday-only appointments, which some viewed negatively against the ease with which chiropractic care could be scheduled in community settings.

Different hours … Some of us can’t get down here at two o’clock because we work, but I would not stop this … Wouldn’t change a thing except for the times. (PTID:88)

[VA clinic] only takes [patients] until 3:30pm, where I could go to an outside chiropractor at 6pm at night, or on a Saturday morning. (PT:167)

##### Scheduling out

Several veterans reported “scheduling out” DC appointments, even if they were not experiencing symptoms. Such appointments were made before activities that might trigger LBP, such as work rotations or training events, and canceled if not needed.

I know it’s extremely difficult to get in and make appointments. I like to try and make appointments one full month out because otherwise, they’d fill up quickly. (PTID:4)

##### Attendance barriers

Additional attendance barriers included the travel distance to the VA chiropractic clinic, vacations, and other health problems.

I see the chiropractor when I can get in … based on my travel problems that I’ve got... Since I bounce back and forth between [two states], it’s sometimes difficult to make an appointment. (PTID:82)

#### Theme 2: Treatment frequency during clinical trial

Most participants were scheduled to receive chiropractic care once weekly, with treatment frequency determined by clinical need and patient scheduling preferences. During the trial, participants had mean attendance of 4.5 visits (range 1 to 7) over 10 weeks. Many participants reported this treatment frequency, or dosage, of chiropractic care was sufficient to address their LBP complaint, although some veterans recommended more or less frequently scheduled treatments.

##### Treatment frequency sufficient for LBP complaint

Many participants reported the weekly treatments received in this study were sufficient to address their primary clinical concern.

Just right. We had good communication and we talked about how I felt. The amount of visits I needed to be seen for versus how much [the chiropractor] thought. We discussed together and made that treatment plan. (PTID:135)

##### More or less frequent treatments desired

Some participants thought frequent treatments, scheduled earlier in the trial, might be beneficial while others wanted less frequent visits.

When we started and [DC] did the first one [treatment], it was great. But then it was a long period before I got to see [DC] the second time. I think more frequently at the beginning of the pain is better than waiting a month later because it almost puts you back where you were. (PTID:88)

I saw [DC] every 2 weeks. I thought it was excessive, but [DC] thought that was what was necessary and [the DC] is the doctor, so I take that advice. (PTID-52)

#### Theme 3: VA chiropractic clinic considerations

Veterans offered many recommendations to impact VA chiropractic clinics, such as hiring additional DCs including female chiropractors, improving the clinic environment, and creating patient-centered treatment visits.

##### Additional VA chiropractors on staff, including female DCs

Several participants recommended VA hire more DCs to improve service delivery within the healthcare system, including female chiropractors for veterans with this preference.

Maybe getting more available? About 2 to 3 weeks before I can get back in with [the chiropractor]. There’s a lot of other people that need to see [chiropractor]. (PTID: 94)

What about female staff? Because a patient like me that has MST [military sexual trauma] and PTSD, I can only work with female providers. There’s a lot of individual needs but if a need is a female provider, make sure that one is available. Make sure you have a balance between male and female participants and male and female care providers. That’s really important … … you don’t want to cause more stress. (PTID:187)

##### Chiropractic clinic environment

Some improvements to the chiropractic clinic environment were suggested, including lighting, room layout, and a more 'healing' atmosphere.

It’s the VA. What that means is it’s more of a hospital setting … I don’t necessarily get that place-of-healing-at-the-VA feeling. Construction projects in the hallway. Random people yelling outside. The room was a little small … huge halogen lights in the office. That bright light, when you’re laying down, if you have a migraine, it’s really hard to deal with. (PTID: 15)

##### Patient-centered treatment visits

Veterans suggested ways to make treatment visits more patient-centered, including providing patient education materials about chiropractic, LBP diagnoses, and self-care strategies, as well as demonstrating how chiropractic equipment works before the manipulation, especially with patients who have anxiety or PTSD.

Me, being who I am, and in the state I am in with PTSD, [treatment] was a lot to get used to, especially using the machine. It makes kind of a weird noise … the only thing I would recommend different is let me hear that gun [chiropractic instrument] before it’s used on my body when I’m lying face down with my eyes closed. That freaked me out. (PTID: 6)

### Domain 2: Selected outcome measures

Descriptive statistics reporting completion times and rates for the outcome measures are reported in the companion article (Long CR, Salsbury SA, Vining RD, Lisi AJ, Corber L, Twist EJ, Abrams T, Wallace RB, Goertz CM: Care Outcomes for Chiropractic Outpatient Veterans (COCOV): a single-arm, pragmatic, pilot trial of multimodal chiropractic care for U.S. veterans with chronic low back pain, Submitted). During interviews, participants reflected upon the content and experience of completing outcome measures (referred to as study forms) during the pilot. Themes discussed questionnaire burden, questionnaire relevance, and the timing and individualization of outcome measures.

#### Theme 1: Questionnaire burden

##### Burden

Many veterans (*n* = 22) stated the study forms were easy to follow and not considered burdensome to complete.

As far as filling out the questionnaires, they were easy. They did not take as long as I thought any of them would take … very minimal burden. (PTID:52)

##### Time-consuming or repetitious

Some individuals said completing the questionnaires was time-consuming, while others commented on the repetitious nature of some measures.

Well, not so much as a burden as much as time consuming and a lot of repetitive questions, just asked a different way or under a different heading I guess. (PTID: 94)

#### Theme 2: Questionnaire relevance

##### Relevance

Most participants agreed the outcome measures were relevant to their experience of LBP. However, some veterans questioned the rationale for including questions about mental health, substance abuse, suicide, or one’s outlook on life.

PTSD and depression … is a huge thing with veterans. But really, I’m going to say it. It gets old, answering the same question every time I go somewhere. ‘Do you feel like killing yourself?’ Well no, I don’t feel like killing myself … I can’t go anywhere in the VA system or take any study … without someone asking me if I feel like killing myself. (PTID:114)

The only one that I was a little bit miffed by or quizzical about was the one about religion. I didn’t know how that fit with back pain. Do I believe in this, that and the other, and I’m going, ‘What does this have to do with my back pain?’ (PTID:85)

#### Theme 3: Questionnaire timing and individualization

##### Timing

Several participants reported that the timing of the outcome measures did not coincide with the presentation or fluctuations in their symptoms:

Answering the questions, sometimes it’s difficult because on that particular day or even that week, my pain might have been a 5 or a 6 where maybe the following week, I didn’t even have a 1 … it’s not a constant thing with me … it’s just hard to put it in a box. (PTID: 111)

##### Individualized approaches

Several people endorsed questionnaires that allow for flexibility or individualization, such as items about activities important to the patient. However, some stated the narrow focus on LBP prevented full evaluation of associated spine-related conditions, such as neck pain. Some suggested adding open field text boxes to allow participants to make personalized comments about their health or study experience.

It asked the 3 things that are important to me. There are some things I want to be able to do that I can’t really do or have a hard time doing … spending time with my 7 year-old son … working on the farm … you’ve got to have all that information to get the proper care and treatment. (PTID: 187)

I was having a lot of problems with my neck and there wasn’t very many questions asking about my neck … If I had a place to make a comment of my own, then I could have told you about my neck. There is no place for me to do that. (PTID: 186)

### Domain 3: Online data collection procedures

Trial data collection procedures highlighted user concerns and technology issues. Most veterans completed all study-related forms online using REDCap without apparent difficulty, although some completed abbreviated outcome measures via a computer-assisted telephone interview. Two veterans mentioned a preference for completing paper forms.

It would be kind of cool if you went and did your therapy, and maybe … I don’t know. If I could have requested it [the questionnaires] on paper. (PTID:4)

#### Theme 1: User concerns

Many participants (*n* = 13) reported little difficulty completing questionnaires using REDCap, noting the email notifications, ease with which surveys could be restarted, and the convenience of using smartphones to complete questionnaires.

It wasn’t difficult. I think every single one I did on my phone … click the button. Take the survey. (PTID:114)

##### Challenges completing online forms

Some participants reported being “locked out” when they missed a data collection window. Others were unsure of questionnaire timing (how often the forms would be completed) or duration (how long each form would take to complete).

I thought I started off strong and yet I did not complete … the online questionnaires on time and therefore I was locked out of those. I feel badly about not completing that part of the survey. (PTID:4)

##### Good computer skills needed

While many participants noted their own “computer savviness,” some wondered how older veterans or persons with low technology skills might fair.

Someone, if they were not really good with technology, they might struggle with it a bit, but I didn’t say it was a problem. (PTID:6)

#### Theme 2: Technology issues

##### Computer or internet access

Eleven participants reported issues completing online forms due to limited internet access in rural locations or personal decisions about computer technology.

Well, I live in the country so sometimes the internet’s sketchy. Sometimes it works, sometimes it doesn’t. (PTID:6)

It’s not that it was difficult for me. I just don’t have a computer at home. I don’t have Wi-Fi. I don’t have internet cable. Where I work, I do. I did my surveys when I was at work. But you know? If I didn’t have that option, I probably wouldn’t have been able to do the surveys at all. (PTID:150)

##### Email invitations going to junk mail

Participants received training on configuring their email to accept REDCap messages, but several reported these emails went to their “junk mail” folders.

I don’t know how you prevent this. I have to go into my junk folder and look for them. Otherwise, I miss them. [Study staff] sent me a text message … reminder … and there was actually an email and a reminder in my junk folder. (PTID:114)

#### Domain 4: Clinical Trial Planning Considerations

Participants described other experiences in this pilot that influenced planning for the full-scale clinical trial. Veterans endorsed established recruitment strategies, such as brochures and having providers introduce patients to studies, and recommended encouraging altruistic service by veterans, increasing veteran awareness about VA chiropractic services, and offering financial compensation. Methods to contact veterans focused on preferences and challenges.

#### Theme 1: Participant recruitment

##### Encourage altruism

Several participants considered their participation in the trial as an opportunity to serve other veterans, especially those with back pain.

I received a call and I figured I could be of use. I was recently out of the service for back problems. (PTID: 162)

##### Awareness of chiropractic services

Some veterans reported not knowing much about the health profession of chiropractic. Others did not know VA offered chiropractic care.

Why I did this study was because I was unaware I could get chiropractic care at the VA. (PTID:150)

The chiropractic. It’s just I haven’t had that experience before. (PTID:137)

##### Financial compensation

Some participants asked how study involvement would be billed or impact travel reimbursement; several appreciated the financial compensation for their time.

[Gift card] is going to buy my hunting stuff … that’s a benefit right there. I mean, that makes you want to do the study. (PTID:167)

#### Theme 2: Communication methods

##### Communication preferences

Participants’ preferred methods of communication with study personnel included telephone (45%) or text message (55%). Most participants reported no communication problems, although 2 reported challenges with contacts by telephone.

I got all of the emails or answering machine, but [study staff] never answers me back … we just couldn’t get together on a time. Wasn’t your fault, wasn’t my fault. It just came together today. (PTID:186)

Veterans were asked about MyHealth*e*Vet (MHV), the VA web-based, personal health record, as a communication tool. Twelve used MHV regularly for visit reminders, provider contacts, record access, and prescription refills. Non-users (*n* = 7) or those unsure if they used MHV (*n* = 4) reported difficulties with internet connection or application functions; password changes; and preferences for receiving appointment reminders by phone.

I signed up because I wanted to get text messages … now I get my appointments texted to me. (PTID:15)

##### Communication barriers

Some veterans reported concurrent enrollment in other VA studies, which was a challenge during interviews or when filling out surveys. A few veterans reported memory, mental health concerns, or other health issues which made communication difficult.

They need to follow up a little bit more, because us veterans are forgetful. I have combat PTSD. I take a lot of meds. And I am forgetful … [staff] need to follow up more with the patient, to get him an appointment. (PTID:167)

## Discussion

VA research agendas call for evaluation studies of non-pharmacological therapies for veterans with chronic musculoskeletal pain, including RCTs of dose, frequency, and duration of CIH treatments, such as chiropractic care [13, 33]. This qualitative study explored veteran perceptions of the research methods and clinical care received in a pilot trial conducted during the planning phase for a full-scale, multi-site, pragmatic RCT of multimodal chiropractic care [31]. RCTs are a well-established methodology for evaluating chiropractic manipulative therapy [34–40], although few prospective RCTs conducted with veteran populations are reported [15]. Further, few publications describe the lessons learned from the planning or implementation of such intensive research projects, with most papers reporting on the development of credible sham procedures [41–43] or patient recruitment [43–46].

Qualitative interviews conducted with the veterans who enrolled in this pilot trial allowed our team to understand potential barriers and facilitators to conducting pragmatic RCTs of chiropractic care in VA settings, findings which may be transferrable to other researchers who plan to conduct clinical trials of chiropractic care and nonpharmacological pain treatments, either within or outside VA healthcare settings. Our pilot study identified critical issues related to treatment scheduling, participant recruitment, and patient communication needs, which we addressed in the development of the full-scale RCT [31]. In addition, these interviews clarified the burden and relevance of selected outcome measures and how best to facilitate online data collection, findings which may help other VA researchers plan their clinical trials, as well as influence how clinicians who deliver manual therapies evaluate patient responses to these treatments. Veterans also offered useful recommendations about the clinic environment within VA and provided insights into the delivery of chiropractic treatments for this patient population, which we describe later in this discussion.

### Treatment scheduling

We sought to understand veterans’ experiences of scheduling and attending VA-based chiropractic visits to plan for a multi-site RCT [47]. Scheduling challenges are common barriers to treatment among veterans, including those enrolled in clinical studies [48, 49]. While most veterans in our pilot reported few scheduling issues, some voiced concerns with wait times for initial visits, limited clinic hours, and a desire for more frequent, or fewer, treatments. Negative perceptions involving VA wait times are well-publicized [50], although a recent study of new patient scheduling for primary care and select specialties reported similar wait times between VA and private sector facilities, with the exception of orthopedic care [51]. In this study, some veterans described “scheduling out” future visits, with cancellations made if a chiropractic appointment was perceived as unneeded. Previous no-shows and appointment age (time since appointment scheduled) are predictors of missed appointments in VA [52]. The number of missed appointments for VA chiropractic visits is unknown. However, patient no-shows may account for 18% of all missed outpatient visits, costing VA upwards of $167 per encounter in 2008 [53]. Our team used information about scheduling patterns and concerns to develop a pragmatic treatment protocol and attendance monitoring plan for the full-scale trial.

### Outcome measures

The NIH Task Force on Research Standards for Chronic Low-Back Pain recommends stakeholder assessment of outcomes of most importance to patients [24]. Veterans in this trial considered most selected outcomes relevant for a study of chronic LBP, with some notable comments. That these veterans approved of personalized questionnaires is consistent with the literature on patient expectations in LBP care and complementary medicine [54–56]. Indeed, recent studies note that patient goals for LBP have only modest alignment with commonly used outcome measures [56, 57]. Some veterans in this pilot trial reported the outcome measure questionnaires were time-consuming or repetitious to complete. Our team expected such comments as we were piloting newer data collection tools against established chronic pain instruments [26, 27]. Based upon this pilot, future clinical trials of chiropractic care in VA should offer streamlined outcome measures to decrease participant burden [24].

Most veterans (95%) enrolled in this study had a documented mental health comorbidity, as was consistent with eligibility criteria that did not exclude persons with these diagnoses from participation, which was one reason our protocol included multiple measures of mental health. Some veterans with mental health conditions may be willing to share de-identified data with researchers [58]. However, others remain concerned about the stigma of mental health diagnoses (depression, PTSD) and military sexual trauma and may be reluctant to engage in treatment [59, 60]. In this trial, veterans often mentioned their specific condition during their interviews. And yet, some negatively viewed research items addressing such topics as alcohol or substance use, anger, and suicide. In addition, the positive outlook subscale of the Healing Encounters and Attitudes List (HEAL), a measure of nonspecific factors in healthcare treatments [61], was considered overtly religious or spiritual by some. Previous research has linked anger with chronic LBP [62] as well as negative spiritual coping or distress with increased mental health diagnoses, symptom severity, and chronic pain [63–65]. Clinical providers, including doctors of chiropractic, should be aware of how common these health concerns are among veterans, institute appropriate assessments, and consider referrals to appropriate specialists for veterans who express emotional or spiritual distress [19, 66, 67].

### Data collection procedures

While most participants successfully completed online data collection using REDCap via computers or smartphones, almost half of those interviewed expressed at least one challenge in doing so. Veterans report satisfaction and competence with using electronic health information technology (e-health), completing online surveys, and engaging in research using web-based applications [68–70]. However, some veterans report computer literacy challenges or experience difficulty using online resources or electronic health records, including MyHealth*e*Vet [70–73]. Our future trial addresses these concerns with additional training on online data collection for participants and tracking protocols for study personnel to assure timely completion of outcome measures.

### Participant recruitment

Our response rates for the overall pilot trial and for this qualitative study (60%) and the low dropout rates for each met our feasibility goals. This pilot evaluated 3 participant recruitment strategies, including personalized letters sent to veterans who were screened through the electronic health record, focused recruitment from the chiropractic clinic, and provider referrals, along with standard techniques such as study-branded brochures [74, 75]. Veterans endorsed these strategies, which proved useful in other VA-based studies of non-pharmacological interventions [76]. Our results also echo the reasons why veterans have participated in clinical studies, including valuing altruism by offering their enrollment as service to other veterans [77]. However, our participants did not identify an altruistic desire to “pay back” healthcare professionals as a recruitment motivator, as identified elsewhere [77]. While monetary compensation was not the primary motivator for enrollment, this finding differs from a study of more recent veterans who reported adequate financial compensation plus the opportunity to help other veterans were key considerations for joining VA-based research [78]. Our full-scale trial incorporated this feedback into the study protocol for participant recruitment and retention [31]. Future studies also may consider social media for veteran recruitment into clinical studies, which may be useful for younger veterans, patients engaged in risky behavior, and those who do not currently use VA-located health services [79].

### VA-based chiropractic services

Participants in this small trial offered broad suggestions for VA-based chiropractic services, which our team used in concert with recent trend analyses [17] to create staffing models that meet patient preferences and protocol parameters for the full-scale RCT [31]. While not all of the concerns mentioned by VA patients can be addressed within such a trial (such as room sizes or provider availability), some suggestions might offer insights for long-term planning of VA chiropractic service delivery beyond this single location and research study [17, 80].

In 2016, onsite VA clinics served over 37,000 unique chiropractic patients and provided nearly 160,000 chiropractic visits [17]. In this qualitative study, veterans recommended additional chiropractic staff to enhance appointment scheduling, which may be challenging as nearly 25% of veterans lack adequate access to health professionals, particularly veterans living in designated Shortage County Areas [81]. Key VA stakeholders have made similar observations about the availability of CIH providers, including chiropractors [82–84]. Improving access to nonpharmacological pain treatments is important, as limited access hampered pain management and increased costs in patients with chronic musculoskeletal pain, especially among younger veterans [85]. Gender-sensitive care, including staffing, is advised by women’s health experts in VA [28, 86]. In this pilot study of chiropractic care, a manually delivered treatment, some participants stated a preference to receive care from a female chiropractor, which currently comprise about 20% of VA chiropractors [17]. Male and female veterans who have experienced military sexual trauma want to choose the gender of their healthcare providers, while many women veterans prefer access to gender distinct clinics and waiting areas [48, 87–89].

Patient preferences for chiropractic clinic environments are not well understood. Key stakeholder groups, especially patients and families, advocate for chiropractic clinics that emphasize comfort, allow for privacy and dignity, and offer ‘healing environments’ [90–95]. Veterans in this pilot described hospital-like environments that might better attend to lighting levels and ambient noise to enhance patient comfort. VA patients also value clinic settings perceived as safe and private [86]. Our participants also requested information about what to expect from a chiropractic treatment, such as the equipment used, anticipated sounds and sensations, and body positioning [19]. Patient education about practices, procedures, and equipment is a commonly reported unmet need among non-users of chiropractic care [96].

#### Methodological rigor and study limitations

Methodological rigor was enhanced through the following strategies [97]. The structured interview guide, interviewer training protocols, peer debriefing with completed transcripts, and previous experience conducting research interviews with patients supported the credibility of these results. Prolonged engagement was established through conducting interviews with multiple participants over the course of the clinical trial. Dependability of findings included the detailed audit trail of coding decisions and extensive use of representative quotes linked to coding themes. Transferability to other VA contexts included data saturation of primary themes across participants, with subthemes included in supplemental tables to identify potential concerns for institutions newly implementing chiropractic care in their settings.

This study has limitations. We did not interview all participants due to scheduling challenges and interviewee non-participation. Those who did not participate, particularly those who self-selected out, may have offered additional perspectives. Veterans who receive chiropractic care in other VA facilities or outside the context of a research study might have other opinions. Data were collected in-person and through telephone interviews, and by study coordinators who were either known or unknown to participants. These procedural differences may have influenced the rapport between participant and interviewer and could have impacted data quality or content. Finally, veterans were not compensated for completing the interview, as they were for quantitative measures, which may have impacted participation. Our team has added financial compensation for participants who complete qualitative interviews in the full-scale trial [31].

## Conclusions

This qualitative study highlighted veteran stakeholders’ perceptions of the feasibility and acceptability of VA-based chiropractic services for the treatment of chronic LBP. Veterans offered important suggestions for conducting a full-scale, pragmatic randomized controlled trial of multimodal chiropractic care in this clinical setting. Key aspects of clinical trial planning addressed through these interviews included defining treatment scheduling protocols, confirming usefulness of multiple recruitment strategies, refining and streamlining outcome measures, enhancing online data collection procedures, and developing multiple means for communication with participants. Veterans also offered suggestions, such as chiropractic staffing considerations, more clinic-like environments, enhanced patient education, including about the availability of chiropractic services in VA, and patient-centered treatment visits which may be useful in administrative decisions about VA-based chiropractic care.

## Supplementary Information

**Additional file 1.** Consolidated Criteria for Reporting Qualitative Studies (COREQ) Checklist

**Additional file 2.** Qualitative Data Tables

## Data Availability

For information about the dataset used in this study, please contact the Office of Data Management and Biostatistics, Palmer Center for Chiropractic Research, Palmer College of Chiropractic, Davenport, Iowa at palmer-research@palmer.edu.

## Abbreviations

CIH: Complementary and integrative health
COCOV: Care Outcomes for Chiropractic Outpatient Veterans (NCT03254719)
COREQ: Consolidated Criteria for Reporting Qualitative Studies
DC: Doctor of chiropractic
DSMC: Data and Safety Monitoring Committee
HEAL: Healing Encounters and Attitudes List
IRB: Institutional review board
LBP: Low back pain
MHV: My Health*e*Vet
MST: Military sexual trauma
NCCIH: National Center for Complementary and Integrative Health
NCRR: National Center for Research Resources
NIH: National Institutes of Health
PASTOR: Pain Assessment Screening Tool and Outcomes Registry
PCP: Primary care provider
PHR: Personal health record
PROMIS®: Patient-Reported Outcome Measurements Information System
PTID: Participant identification number
PTSD: Post-traumatic stress disorder
RCT: Randomized controlled trial
REDCap: Research Electronic Data Capture
SD: Standard deviation
US/USA: United States of America
VA: U.S. Department of Veterans Affairs, Veterans Health Administration
VERDICT: Veteran Response to Dosage in Chiropractic Therapy (NCT04087291)
VISN: Veterans Integrated Service Network (US)
