# Supplementary material for: Care Outcomes for Chiropractic Outpatient Veterans (COCOV): a qualitative study with veteran stakeholders from a pilot trial of multimodal chiropractic care
Source: Pilot Feasibility Stud. 2022 Jan 14;8:6. doi: 10.1186/s40814-021-00962-5 (PMC8759237; doi:10.1186/s40814-021-00962-5)
Supplement: Supplementary file 1 — Additional file 1. Consolidated Criteria for Reporting Qualitative Studies (COREQ) Checklist [file 40814_2021_962_MOESM1_ESM.docx]

Supplementary Materials - Consolidated Criteria for Reporting Qualitative Studies (COREQ) Checklist

| **Domain 1: Research Team and Reflexivity** | | |
| --- | --- | --- |
| **Personal Characteristics** | | |
| Interviewer/ Facilitator | Who conducted interviews? | Janice Hubbard, DC, MS  Elissa Twist, DC, MS |
| Credentials | What were the researchers’ credentials? | Salsbury - PhD-prepared qualitative researcher with expertise in clinical trials of complementary and integrative health interventions  Twist - Doctor of chiropractic and MS-prepared clinical research specialist with experience conducting patient interviews in clinical trials  Wallace - Medical doctor and epidemiologist with expertise in clinical trials of complementary and integrative health interventions  Vining - Doctor of chiropractic with expertise in chiropractic clinical practice and clinical trials in military and veteran health settings  Goertz - Doctor of chiropractic and health services researcher with expertise in clinical trials in military and veteran health settings  Long - Biostatistician and clinical trialist with expertise in military and veteran health settings |
| Occupation | What were the researchers’ occupation(s) at time of study? | Salsbury, Vining - Clinical research faculty at Palmer Center for Chiropractic Research  Goertz - Vice Chancellor of Research and Health Policy at Palmer College of Chiropractic  Long - Director of Research and the Office of Data Management and Biostatistics at Palmer Center for Chiropractic Research  Twist - Study coordinator at Palmer Center for Chiropractic Research  Wallace – Professor, The University of Iowa College of Public Health |
| Gender | Researcher genders? | Research team included members who identified as female and male genders. |
| Experience/ Training | Experience and training of researchers? | The 2 interviewers (Twist, Hubbard) were experienced study coordinators who had completed patient interviews as part of previous clinical studies. Interviewers received one-on-one training from the lead qualitative research (Salsbury) on the interview protocol, interview question schedule, and best practices for conducting qualitative interviews. Follow-up training was provided after completion of initial interviews based upon quality control checks of transcripts to address areas for improvement, largely consisting of follow-up/probing strategies. |
| **Relationship with Participants** | | |
| Relationship Established | Researcher established relationship with participants before start of study? | One interviewer (Hubbard) had considerable contact with most participants due to her involvement in enrollment procedures and ongoing contact during the trial. The second interviewer (Twist) mostly engaged in telephone contact with participants during the trial, although had some face-to-face interactions with some participants. The data analyst (Salsbury) and other investigators did not have an established relationship with the patients who participated in this study. |
| Participant Knowledge of Interviewer | What did participants know about researchers? | Participants had minimal knowledge of the interviewers, including their role in the study and institutional affiliation. Participants knowledge of the investigative team was limited to information provided in the informed consent document. |
| Interviewer Characteristics | What researcher characteristics reported to participants? | Role, training, professional credentials, workplace, experience in clinical research. |
| **Domain 2: Study Design** | | |
| **Theoretical Framework** | | |
| Methodological Orientation & Theory | Methodological orientation underpins study? | This nested qualitative study within a single-arm, pragmatic clinical trial used a descriptive, phenomenological perspective to understand veterans’ experiences as participants in the trial and as patients within VA-based chiropractic clinics. |
| **Participant Selection** | | |
| Sampling | How were participants selected? | All clinical trial participants were invited to complete qualitative interviews at the conclusion of their involvement in active care. |
| Method of Approach | How were participants approached? | Participants were introduced to the interview during the informed consent process, with all completing designated forms to allow audiorecording of the interview. Towards the end of active care, study coordinators contacted participants by telephone to arrange a data/time and method (in-person or by telephone) to complete the exit interview. |
| Sample Size | How many participants were in the study? | 24 of 40 clinical trial participants completed exit interviews for a response rate of 60%. |
| Non-participation | How many people refused to participate or dropped out? Reasons? | Of the 16 participants who did not complete an exit interview, 5 participants were not able to be contacted by study staff. The other non-participants opted not to complete interviews. |
| **Setting** | | |
| Setting of Data Collection | Where was the data collected? | Face-to-face in hospital consultation rooms (n=11) or via telephone interview (n=13). |
| Presence of Non-participants | Was anyone else present besides the participants and researchers? | In some cases, a family member was in attendance during interview per the request of the participant. |
| Sample Description | What are the important characteristics of the sample? | Results – Sample characteristics: Of 40 trial participants, 24 completed interviews (60% response). Male veterans (n=16; 67%) predominated the interviews, although the inclusion of 8 women veterans (33%) achieved our goal of a minimum 20% female sample. Mean age (SD) was 51.7 (15.7) years with most participants stating their race as white (88%) and their ethnicity as non-Hispanic or Latinx (96%). All participants (100%) stated cLBP was an ongoing problem for more than 6 months. |
| **Data Collection** | | |
| Interview Guide | Were questions, prompts, guides provided by the authors? Was it pilot tested? | Table 1. provides the interview guide. Semi-structured questions were designed to understand the participant experience in the clinical trial and gather recommendations about chiropractic care within VA. The interview schedule was reviewed by the investigative team and clinical partners, but not pilot-tested before use with patients. |
| Repeat Interviews | Were repeat interviews made? | No repeat interviews were conducted. |
| Audio/visual Recording | Did research use audio or visual recording to collect the data? | Audiorecorded with digital recorders. |
| Field notes | Were field notes made during and/or after the interview? | No fieldnotes were made during or after the interview session. |
| Duration | What was the duration of the interviews? | Patient interviews lasted between 15 to 30 minutes in an effort to decrease participant burden. |
| Data Saturation | Was data saturation discussed? | Credibility and completeness of findings noted in the discussion section. |
| Transcripts Returned | Were transcripts returned to participants for comment and/or correction? | Transcripts were not returned to participants for comment and/or correction. |
| **Domain 3: Data Analysis and Findings** | | |
| **Data Analysis** | | |
| Number of Data Coders | How many data coders coded the data? | 1 coder (Salsbury) coded the data. Co-investigators reviewed coding structure in data management spreadsheets. |
| Description of Coding Tree | Did authors provide a description of the coding tree? | The coding tree with superordinate (domains) and subordinate (themes and subthemes) categories are described in the data analysis section of the methods and outlined in Table 2, with representative quotes linked to each theme offered in Supplementary Materials. |
| Derivation of Themes | Were themes identified in advance or derived from the data? | Themes were derived inductively from the data during analysis. |
| Software | What software, if applicable, was used to manage the data? | Not applicable; data were managed using spreadsheets. |
| Participant Checking | Did participants provide feedback on the findings? | Participants did not provide feedback on the findings. |
| **Reporting** | | |
| Quotations Presented | Were participant quotations presented to illustrate themes / findings? Was each quotation identified? | Representative quotations are used to illustrate all themes in both the text of the results and in supplementary materials. All quotes presented by participant identification number. |
| Data/Findings Consistent | Was there consistency between the data presented and the findings? | Yes. In addition, supplementary materials provide evidence of consistency between at and reported findings. |
| Clarity Major Themes | Were major themes clearly presented in the findings? | Yes, in written narrative, table, and supplementary materials. |
| Clarity Minor Themes | Is there a description of diverse cases or discussion of minor themes? | Yes, in written narrative, table, and supplementary materials. |

<https://academic.oup.com/intqhc/article/19/6/349/1791966/Consolidated-criteria-for-reporting-qualitative>
