# Supplementary material for: Care Outcomes for Chiropractic Outpatient Veterans (COCOV): a qualitative study with veteran stakeholders from a pilot trial of multimodal chiropractic care
Source: Pilot Feasibility Stud. 2022 Jan 14;8:6. doi: 10.1186/s40814-021-00962-5 (PMC8759237; doi:10.1186/s40814-021-00962-5)
Supplement: Supplementary file 2 — Additional file 2. Qualitative Data Tables [file 40814_2021_962_MOESM2_ESM.docx]

| **Additional File 2. Table 1. DOMAIN 1 - Chiropractic Service Delivery for a Clinical Trial in Veteran Affairs (VA) Healthcare Facilities** | |
| --- | --- |
| **Theme 1: Appointment Scheduling Process for VA Chiropractic Care** | |
| ***Subtheme*** | **Representative Quote (Patient Identification)** |
| *Clinic hours limited for chiropractic appointments* | - I have a full-time job. I usually work until 4:30 or 5 o’clock. The latest appointment I was able to get any day was 2:30. I had to leave work early once a week for the past 7 weeks to be able to receive treatment. I work for a really good company. They are helping me through this. But I think for other people that could be an issue. (PTID:114) - Different hours. It would improve definitely under different hours. Some of us can’t get down here at two o’clock because we work, but I would not stop this…Wouldn’t change a thing except for the times. (PTID:88) - [VA clinic] only takes [patients] until 3:30pm, where I could go to an outside chiropractor at 6pm at night, or on a Saturday morning. (PT:167). |
| *“Scheduling out” future appointments* | - I had a drill right after and [DC] actually scheduled for additional [visit] right after the drill so that I would be able to come in and follow-up because [DC] knew that was the time that I usually injured my back. (PTID:29) - I know it’s extremely difficult to get in and make appointments. I like to try and make appointments one full month out because otherwise, they’d fill up quickly. (PTID:4) - I need to stretch out [appointments]. I just feel like at the time I was just feeling good, I was back in a lot of pain. So I pre-booked. (PTID:94) - I asked if I felt like I didn’t need that appointment, could I cancel it and [chiropractor] said ‘no problem’. Yes, I can. I just didn’t have the appointments, cancel them and let somebody else get in and if I need it, I got it. (PTID:186) |
| *Barriers to treatment attendance* | - I see the chiropractor when I can get in…based on my travel problems that I’ve got...Since I bounce back and forth between [two different states], it’s sometimes difficult to make an appointment. (PTID:82) - There was one week where I was out of town for work. And there was another week where he was on vacation that we didn’t see each other. But, for the most part, I was able to get my appointments scheduled. (PTID:114) - Other health issues that I’ve had have caused my need to reschedule [chiropractic appointments]. (PTID:141) |
| **Theme 2: Chiropractic Treatment Frequency During Clinical Trial** | |
| ***Subtheme*** | **Representative Quote (Patient Identification)** |
| *Treatment frequency considered sufficient for back pain complaint* | - I’d say [I had] the right amount of visits. (PTID:162) - It was probably just about the right amount of times. And they were very helpful in adjusting that to the days that I could make it. (PTID:163) - Just right. We had good communication and we talked about how I felt. The amount of visits I needed to be seen for versus how much [chiropractor] thought. We discussed together and made that treatment plan. (PTID:135) - I see the chiropractor when I can get [there], based on my travel problems that I have…Just getting in to see the chiropractor and trying to make it a steady routine, instead of a spotty one, which has happened. I think on a weekly basis it was just about right. (PTID:82) - It was good. No, it was about right. (PTID:13) |
| *More or less frequent treatment desired* | - When we started and [the chiropractor] did the first one [treatment], it was great. But then it was a long period before I got to see [chiropractor] the second time. I think more frequently at the beginning of the pain is better than waiting a month later because it almost puts you back where you were. (PTID:88) - When you’re first seeing the chiropractor, you tend to want to see them 6 days a week. You know? It’s like, fix me! Fix me! (PTID:111) - I would always love to keep going with chiropractic care because of the maintenance, but I also know that if you do that to the VA [healthcare facility], then it might limit someone else’s ability to get help. (PTID:15) - I’m not real familiar with chiropractic services. I mean in my case, when I’m having issues, then yes, maybe seeing them more often would be helpful, but I’ve never asked to do that, so if that’s the case, that’s my own fault. But as a rule, it’s just fine. (PTID:141) - I saw [chiropractor] every 2 weeks. I thought it was excessive, but [chiropractor] thought that was what was necessary and [the chiropractor] is the doctor, so I take that advice. (PTID-52) |
| **Theme 3: Personnel Staffing in VA Chiropractic Clinics** | |
| ***Subtheme*** | **Representative Quote (Patient Identification)** |
| *Additional VA chiropractors on staff* | - Sometimes there’s a wait. We only have so many chiropractors here…[The chiropractor] is spread pretty thin. So, hire more chiropractors! Subliminal! (PTID:4) - Maybe getting more available? About 2 to 3 weeks before I can get back in with [chiropractor]. There’s a lot of other people that need to see [chiropractor]. (PTID:94) - Increase the number of chiropractors. Increase the number of doctors and nurses. All of your budgeting issues, I understand, and hiring issues. Hopefully the new director of the VA is going to be able to take care of some of that...If there were six or seven chiropractors, it would make things easier. (PTID:82) - If you’ve got to wait a month for a chiropractor, that’s not good. (PTID:88) - It’s just you guys [chiropractors] are overworked. You just have to be patient and everything, but like with all the other doctors and everything, you guys are just overworked. (PTID:137) |
| *Female chiropractors needed on VA staff* | - A patient like me, that has MST [military sexual trauma] and PTSD [post-traumatic stress disorder], I can only work with female providers. There’s a lot of individual needs, but, if I need a female provider, make sure that one is available…Make sure you have a balance between male and female participants and male and female care providers. That’s really important…you don’t want to cause more stress. (PTID: 187) - I was very happy to a female option. I’ve always wanted it, a female doctor, a female chiropractor, a female everything. If I went in socks, and things, of course, a man-doctor understands that, but I just felt more comfortable with her. So, I’m glad that I had that option. I think she did a great job.” (PTID:15) |
| **Theme 4: Other Considerations for Clinical Trials in VA Chiropractic Clinics** | |
| ***Subtheme*** | **Representative Quote (Patient Identification)** |
| *Chiropractic clinic environment and information about treatment* | - It’s the VA. What that means is it’s more of a hospital setting…I don’t necessarily get that place-of-healing-at-the-VA feeling. Construction projects in the hallway. Random people yelling outside. The room was a little small… huge halogen lights in the office…that bright light, when you’re laying down, if you have a migraine, it’s really hard to deal with. (PTID:15) - More in depth and more informative as to what my problem is, what I can do about it and so on… Not knowing the science of chiropractic, I don’t know if it [treatment] was enough or not. (PTID:85) - Me being who I am, and in the state that I’m in with PTSD…It was a lot to get used to, especially using the machine that she uses. It makes kind of a weird noise…With that being said, the only thing that I would recommend different is let me hear that gun [adjusting instrument] before it’s used on my body when I’m lying face down with my eyes closed. Because that freaked me out. (PTID:6) - Continue to encourage students. Students to come in and participate in continuing their education as far as working with [chiropractor]. I think that’s a wonderful idea for the VA to allow that to happen. And the provider that you have right now is a great person for them to work with. (PTID:135) |
| *Chiropractic treatment in clinical trials for back pain* | - Within the study, it was just a focus on my lower back when the whole back interacts as one piece…It’s hard to explain. Make sure that the whole back and neck and everything that is connected is taken care of. I actually had to get another referral for [the chiropractor] to treat the rest of my back. But it needs to be a full treatment because the alignment and everything. If you only treat one section, of course, it is going to keep going out of whack, if you don’t treat the rest of what’s connected. (PTID:187) - I was kind of a disappointed, as it didn't do the upper back, but that's fine....the chiropractor just doing the upper back a little bit better, and the shoulders areas where it was bothering me. I don't think it would have been that much more to do that while I was there. (PTID:13) - As far as the chiropractic care I received, I was unsatisfied. I could not tell if the chiropractor was holding back treatment due to a study, I could not tell if he was told to do very, very minimal treatment. (PTID:52) - I did something in between my shoulder blades. I popped something out of place. And the chiropractor doesn’t seem to want to make it cavitate. Doesn’t want to make it go pop, just more or less massages that part. Pushed on it a few times, but…she hasn’t made it pop. (PTID:111) - The very first instance [chiropractic manipulation], I was not prepared for that sharp snap and it really hurt. After that, [treatment] hasn’t really been that bad. (PTID:29) |

| **Additional File 2. Table 2. DOMAIN 2 - Veteran Perceptions of Selected Outcome Measures for a Chiropractic Clinical Trial in Veterans Affairs Healthcare Facilities** | |
| --- | --- |
| **Theme 1: Questionnaire Burden** | |
| ***Subtheme*** | **Representative Quote (Patient Identification)** |
| *Questionnaires were not burdensome* | - As far as filling out the questionnaires, they were easy. They did not take as long as I thought any of them would take…very minimal burden. (PTID:52) - [Answering] wasn’t terrible, but it wasn’t something I was wanting to do every single day of my life. (PTID:6) - [Questionnaires] were easy. I don’t think it was a burden at all. (PTID:135) - No work. No burden whatsoever. (PTID:137) |
| *Questionnaires were time-consuming or repetitious* | - No particular burden. It takes some time. That’s it. And, you know, I got the time. (PTID:82) - I kind of felt sometimes when I was doing the study on the computer, some of the stuff seemed like it was repetitive and I’m sitting there thinking to myself, ‘Why [are] they asking me this again, they just asked this?’ So maybe they [the researchers] need to look at that a little bit. (PTID:37) - Well, not so much as a burden as much as time consuming and a lot of repetitive questions, just asked a different way or under a different heading, I guess. (PTID: 94) - I felt like sometimes I was asked the same question a bunch of different ways. (PTID:15) - On the questions that I got periodically…say for example, the pain in my back. The first couple of them were fine with the pain in my back. After that, the pain in my back was gone and everything was okay, fine and it still is today. But when you keep asking the same questions about that, you’re answering the same, you’re not really feeling like you’re putting anything out to it. It’s all the same. It’s a repeat. (PTID:186) - It was time-consuming, and that was the biggest burden. (PTID:141) - It seems a little bit redundant maybe, but then again they’re all asked in different aspects in different ways, so you’re trying to get… rather than just asking somebody something once you ask them a different way and get a better answer out of it, so it’s interesting. (PTID:111) |
| **Theme 2: Questionnaire Relevance** | |
| ***Subtheme*** | **Representative Quote (Patient Identification)** |
| *Questionnaires relevant for back pain trial* | - They were pretty poignant and specific questions. You were able to gauge specifically to what you, individually, perceived…because a lot of them were on a scale. So it was easier than a yes/no question. That makes it easier, because it is not always one or the other. It was a good set of questions. (PTID: 135) - I’ve done enough surveys over the years that I understand where a lot of the questions come from. Not necessarily are they specific to the back. Some of them are whole person concepts, just a general feeling of how you’re doing. Then, how can the back contribute to that, one way or the other. (PTID:82) - The forms are ok…I could see how they related. (PTID:16) - I think they all had something that applied to me. You can’t know everything about them. Different people are different, so you don’t know what you need to ask me. (PTID:111) - Everything related in some way, shape or form. Like I said, your physical, mental and emotional health all kind of run together. I mean they ask you questions about pretty much every aspect. (PTID:150) |
| *Questionnaire topics considered irrelevant, intrusive, or unclear* | - You know, PTSD and depression and all that stuff is a huge thing with veterans. But really, I’m going to say it. It gets old, answering the same question every time I go somewhere. ‘Do you feel like killing yourself?’ Well no, I don’t feel like killing myself… I can’t go anywhere in the VA system or take any study…without someone asking me if I feel like killing myself. (PTID:114) - Some of the questions like under the post-traumatic stress disorder like asking me if I try not to think about you know. I had an accident when I was 15, which has impacted me a lot. (PTID: 94) - I kind of thought it was interesting that they had psychological questions. As far as mental health…I can’t say that my back is causing me a mental health problem, although I do suffer from depression…I think that that can be a big thing and especially if you’re trying to work and you just can’t. (PTID:111) - Some of the questions, I questioned. Like the anger. The stuff like that. If you’ve ever noticed you’re angry because of [your] back…Well, no. What does this have to do with my back? (PTID:167) - The only one that I was a little bit miffed by or quizzical about was the one about religion. I didn’t know how that fit with back pain. Do I believe in this, that and the other, and I’m going, ‘What does this have to do with my back pain?’ (PTID:85) - That I didn’t answer. The religious aspect…turned me off right then and there. (PTID:137) |
| **Theme 3: Questionnaire Timing and Individualization** | |
| ***Subtheme*** | **Representative Quote (Patient Identification)** |
| *Questionnaire timing did not match symptom presentation* | - The questions never seem to come when you’re actually in pain, more like after the fact…it depends on what you are feeling at the time you’re taking the questionnaire…If you’re feeling terrible, you’re going to get negative answers all the way. If you’re feeling great, you’re going to get fairly good answers. (PTID:88) - Answering the questions, sometimes it’s difficult because on that particular day or even that week, my pain might have been a 5 or a 6 where maybe the following week, I didn’t even have a 1…it’s not a constant thing with me…it’s just hard to put it in a box. (PTID: 111) - I am in excruciating pain right now, but not normal for me. It’s good timing you called today. We finally have a back issue I can address at the time we need to address it. (PTID: 141) |
| *Individualized approaches to questionnaires useful or needed* | - Flexibility is key for me. It asked about all the important things to me that I would want other people to be asked. There was a lot of questions about substance abuse and all that. I’m not a person that wants you to ask those questions…I’m not in a situation that some of those people are…so I want you to ask them, but I didn’t think they were relevant to me. (PTID:15) - Those questions are for your general people. Some people do drink a lot or do drugs or smoke a lot. I don’t do any of those. At least I’ll have a drink or two here and there but not on a regular basis. (PTID:94) - It asked the 3 things that are important to me. There are some things I want to be able to do that I can’t really do or have a hard time doing…spending time with my 7 year-old son… working on the farm…you’ve got to have all that information to get the proper care and treatment. (PTID: 187) - Topics of my welfare and being able to actually do some of the things that I always enjoyed a bit more…like going out and mowing my grass and going fishing and things like that. (PTID: 163) - I was having a lot of problems with my neck and there wasn’t very many questions asking about my neck…If I had a place to make a comment of my own, then I could have told you about my neck. There is no place for me to do that. (PTID: 186) - I think that [communication] could be more emphasized…not just the care itself but the doctor-patient relationship. Are they treating you fairly, you know? (PTID: 114) - The questionnaires were pretty much all kind of about the same. But I felt like I could give a little bit more detail… like I got to give more information about how I felt, like [chiropractic] was kind of really benefitting me. (PTID:150) |

| **Additional File 2. Table 3. DOMAIN 3: Veteran Perceptions of On-line Data Collection Procedures for a Chiropractic Clinical Trial in VA Healthcare Facilities** | |
| --- | --- |
| **Theme 1: User Concerns with On-line Data Collection** | |
| ***Subtheme*** | **Representative Quote (Patient Identification)** |
| *Little difficulty completing forms on-line* | - I had no trouble at all. [Email] came up and said I had one [questionnaire] to do and I just clicked on it and it was there…there was times when I was doing these studies where I would be in the middle of it [a questionnaire] and it went away and I would be like, ‘Oh no! Now what do I do? How do I get it back? Do I start it all over again or what?’ I just clicked on it again and waited a bit and then it would be right where I left off again. (PTID:37) - The surveys and the emails? Every time I clicked on, they worked. (PTID:187) - It wasn’t difficult. I think every single one I did on my phone…click the button. Take the survey. (PTID:114) - The easiest part of it all. It came up and yes, as far as response and everything in it, just kind of bang, bang, bang, it was there. When you answer the last question which says…the last question, you close it and you’re done. (PTID:186) - No. Not much of a challenge. I think it was a very easy to get to. (PTID:163) |
| *User challenges completing on-line forms* | - I thought I started off strong and yet I did not complete…the online questionnaires on time and therefore I was locked out of those…I did not get to do the really long ones that got more in detail and more in-depth…that might have been more of a burden. I feel badly about not completing that part of the survey. (PTID:4) - Were those the online questionnaire things I was supposed to do? I forgot to do half of them but I don’t think they were difficult when I did them…it was more just when I would check my email on my phone, I wouldn’t go do it and then I’d forget about it. (PTID:7) - I try to be vigilant about answering all the questions, the surveys but I don’t know. (PTID:94) - I actually missed the very first one… By the time I was able to get onto it that the time had expired for the first one. And I was all like, ‘Oh my gosh. Am I kicked out of the study? I couldn’t do the thing in time.’ (PTID:150) - I wouldn’t say anything to make it easier because like I said, I didn’t really get to do the survey questions. (PTID:162) - I don’t think there was any difficulty. It was more just when I would check my email on my phone, I wouldn’t go do it [questionnaire] and then I’d forget about it. (PTID:29) - It would be kind of cool if you went and did your therapy, and maybe…I don’t know, if I could have requested it [the forms] on paper. (PTID:4) - I don’t think it was that bad. It was just they [survey invitations] popped up all of a sudden…it was coming in at weird times…I didn’t have too much trouble, just that they popped up out of the blue. (PTID:88) - They got easier as it went along…the next one that I got seemed that it took me a couple of hours. I don’t know. I did it for three days. I didn’t know if it was ever going to end…Maybe it’s because of my first experience, I didn’t know what I was going to have to answer, but it seemed like a long time. (PTID:111) |
| *Good computer-skills might be needed* | - Someone, if they were not really good with technology, might struggle a bit, but I didn’t say it was a problem. (PTID:6) - [Online data collection] could be difficult, you know, for some. I am sure that I am one of the younger veterans in this study. You get some of these older vets that aren’t as tech savvy? That might become difficult for them. (PTID:114) - [Questionnaires] are on the computer. How hard can it be? A lot of your 60 plus years and older are going to be kinda iffy. I am 50 and I am awesome with a computer. So, around my age, or younger, are going to be the ones you will probably hit pretty good with a computer. (PTID:167) - Now there was difficulty in filling out one of the forms. I didn’t learn how to do it until after. But I had to select an a, b, c, d type thing and/or a yes, no type thing and I’d select something but it would go to something different. It was too small. So I gave up on that. (PTID:137) - Not too difficult. I think… I am not an internet person. I don’t like getting online…But I mean I have an email account just specifically for those kinds of purposes. Because I am not on the internet. (PTID: 150) |
| **Theme 2: Technology Issues with On-line Data Collection** | |
| ***Subtheme*** | **Representative Quote (Patient Identification)** |
| *Computer or internet access issues* | - I live in the country so sometimes the internet’s sketchy. Sometimes it works, sometimes it doesn’t. (PTID:6) - The second one that I missed, I did not have access to a computer at that time. (PTID:4) - It’s not that it was difficult for me. I just don’t have a computer at home. I don’t have Wi-Fi. I don’t have internet cable. Where I work, I do. I did my surveys when I was at work. But you know? If I didn’t have that option, I probably wouldn’t have been able to do the surveys at all. (PTID:150) |
| *Email invitations went to junk mail* | - You told me to check the junk mail. It went to junk mail every time, so I had issues with that. (PTID:5) - I don’t know how you prevent this. But I actually have to go into my junk folder and look for them. Otherwise, I miss them. [Project manager] sent me a text message…reminder…and there was actually an email and a reminder in my junk folder. (PTID:114) |

| **Additional File 2. Table 4. DOMAIN 4: CLINICAL TRIAL PLANNING CONSIDERATIONS** | |
| --- | --- |
| **Theme 1: Participant Recruitment Through Engagement of VA Patients and VA Health System** | |
| ***Subtheme*** | **Representative Quote (Patient Identification)** |
| *Encourage research participation as an altruistic service to other Veterans* | - I received a call and figured I could be of use. I was recently out of the service for back problems. (PTID: 162) - I just wanted to participate and see how that [chiropractic] worked. But also, to contribute to the research…so that it can better assist people in the future. (PTID: 135) - It’s nothing to answer a few questions, every couple weeks, and potentially help out future veterans...I have quite a few friends with back pain…it’s definitely an issue that needs to be looked into more for veterans. (PTID: 114) - I like being in studies and trying to make a difference and, as I have the opportunity to make a difference in people’s lives while getting care, then that’s a plus. (PTID: 187) - There are quite a lot of vets that actually work at the VA that have back problems. Just touch base with them and see if they want to get into the study. (PTID:167) |
| *Veteran awareness of chiropractic care or its availability in VA* | - Why I did this study was because I was unaware I could get chiropractic care at the VA. (PTID:150) - Well, it sounds a little strange, but I kind of felt like, well, why not? Maybe sometimes it [chiropractic] could help, or we’ll find out if this is a treatment that can help when I do have [back] problems. (PTID:141) - The chiropractic. It’s just I haven’t had that experience before. (PTID:137) |
| *Offer financial compensation for time and travel* | - The gift cards were a nice enticement, too, but I would’ve done [the study] without a doubt. (PTID:82) - Obviously, the [gift card] is going to buy my hunting stuff… So, that’s a benefit right there. I mean, that makes you want to do the study. (PTID:167) - You said you were going to reimburse my travel? (PTID:13) - This is coming from me, as a veteran. You can’t really persuade us to do something we don’t want to do… there was a gift card incentive…I know that’s probably nice for people. I am going to admit it is nice to receive that as part of this. But that did not incentivize me in any way to take part in this study. (PTID:114) |
| **Theme 2: Communication with Study Participants** | |
| ***Subtheme*** | **Representative Quote (Patient Identification)** |
| *Communication preferences, including telephone and MyHealtheVet (MHV)* | - They called me and reminded about my appointments all the time. (PTID:37) - I did try to call and leave a message with the study personnel and asked them to call me back a few times and they didn’t get back to me. So, I was disappointed in that. I had a question about surveys and if I had missed one. (PTID:187) - I got all of the emails or answering machine, but [study staff] never answers me back…we just couldn’t get together on a time. Wasn’t your fault, wasn’t my fault. It just came together today. (PTID:186) - I signed up because I wanted to get text messages…now I get my appointments texted to me. (PTID:15) - I use it [MHV] to refill and track my prescriptions. I use it to contact providers. I look at my medical notes and that type of thing. (PTID:135) - When I originally went to VA, I got a big packet of information. There was a pamphlet in there on the My Health*e*Vet…I have only been going through the VA now for maybe 6 months. I am sure there is a wealth of knowledge for me to look at. (PTID:114) - I haven’t used it in a while. I was just looking at some paperwork that reminded me I need to check and see if my account’s still active or sign back out. (PTID:82) - I don’t use that too much… Seems like they always want to change the password all the time even though I’ve been in it. (PTID:85) - No. I’m not familiar with that. I’m not using that online so much. I don’t know if that’s the same thing as when I call the telephone to reorder prescriptions or not; probably not. (PTID:111) |
| *Potential communication challenges, including veteran health status, cognitive concerns, and enrollment in multiple studies* | - I honestly could not remember [the questionnaires used in this study]. (PTID:162) - You’re asking about things that there’s no way my brain would remember… I don’t remember being upset or complaining…I don’t remember what this is…I know it has something to do with chiropractic, but I honestly don’t know why I’m here. (PTID:29) - I think they need to follow up a little bit more, because us veterans are forgetful. I mean I have combat PTSD. I take a lot of meds. And I am forgetful…[staff] need to follow up more with the patient, to get him an appointment. (PTID:167) - Which study was this again, the first one? The chiropractic, okay. I thought you said [health tracker brand name] for a second there. (PTID:94) - It just depends on how your back feels I think. It definitely puts you out… I should bring my wife in and let you talk about this. My wife understands when I’m out and when I’m focusing on my family versus I’m focusing on something else. (PTID:12) |
